# Absorbable String-Loop Suture for Closing a Gastric Defect Following Endoscopic Full-Thickness Resection

**DOI:** 10.1055/a-2933-9577

**Published:** 2026-08-25

**Authors:** Shibo Song, Xiaolong Rao, Haowa Wu, Yuan Tian, Long Rong

**Affiliations:** 1 Endoscopy Center 26447 Peking University First HospitalBeijingBeijingChina

## 



**Video 1** Successful closure of a gastric defect following endoscopic full-thickness resection using the absorbable string-loop suture.


**Fig. 1:**
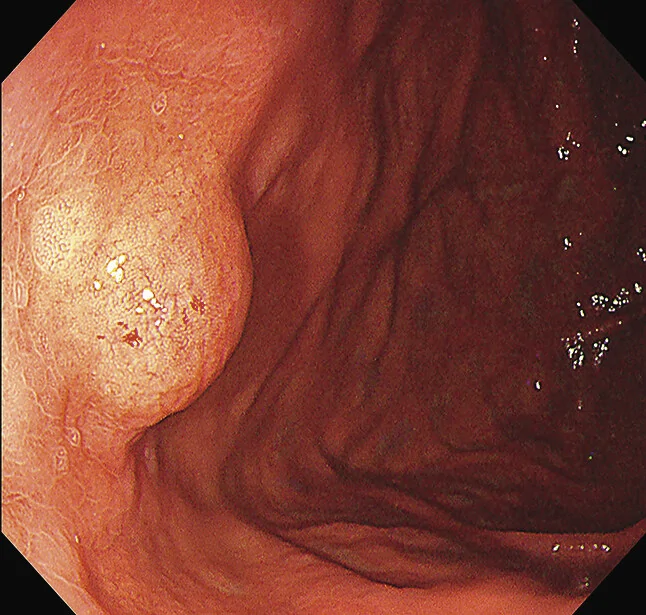
A submucosal tumor on the posterior wall of the gastric fundus during gastroscopy.

**Fig. 2:**
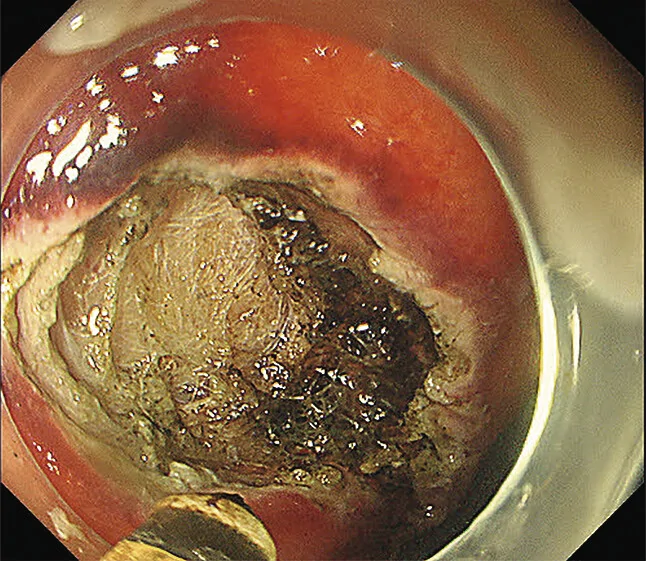
Gastric defect after endoscopic full-thickness resection, with typical yellowish, lobulated retroperitoneal adipose tissue visible at the base.

**Fig. 3:**
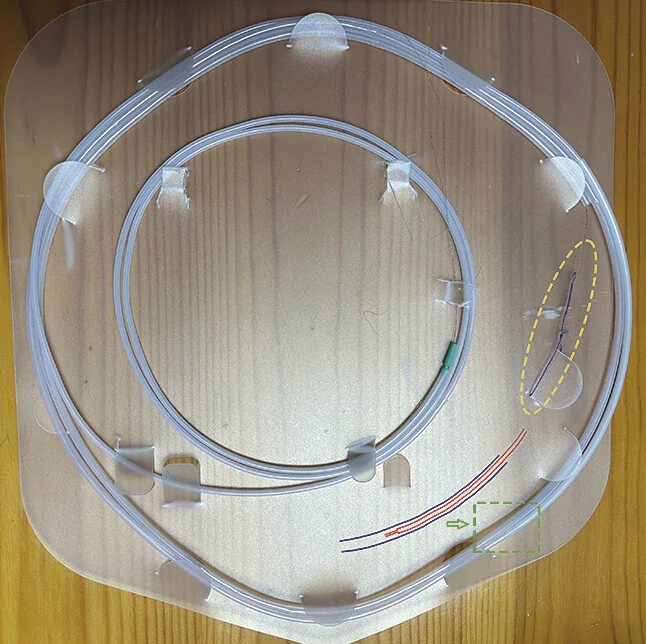
Modified prototype of the absorbable string-loop suture. Notes: The suturing device comprises three components: (1) a purple absorbable closure loop with a pre-tied sliding knot; (2) a spotted fine traction thread; and (3) two transparent nested polytetrafluoroethylene sheaths. Color-coded indicators highlight critical structures: yellow dashed circles for the closure loop and its junction with the traction thread; green dashed boxes for the dual-sheath overlap region; and blue/red solid lines for the schematic diagram of this overlapping section.

**Fig. 4:**
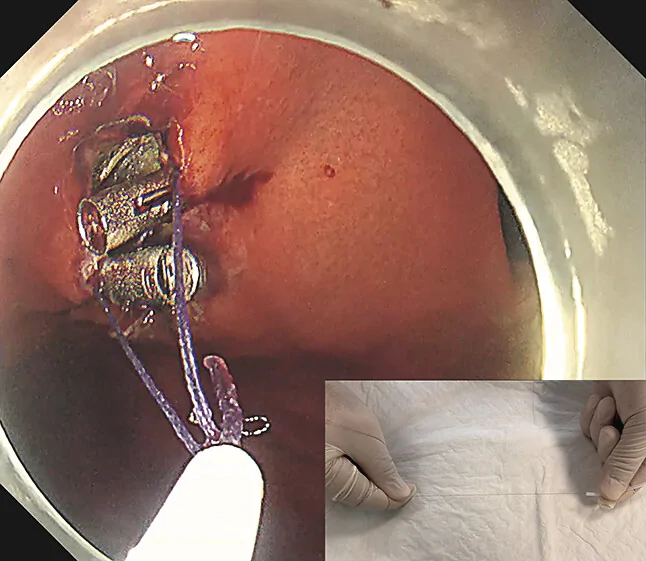
The inner sheath was used to advance the pre-tied sliding knot to tighten the string-loop.

**Fig. 5:**
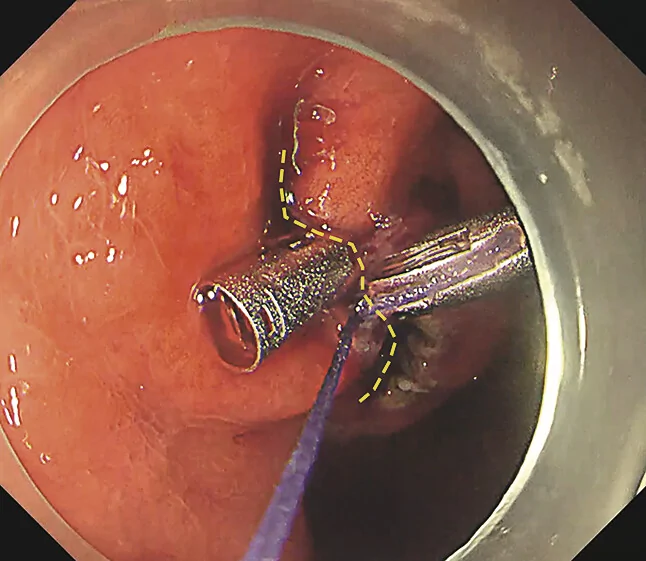
The defect drawn into a linear configuration (yellow dashed line) by the string-loop suture.

**Fig. 6:**
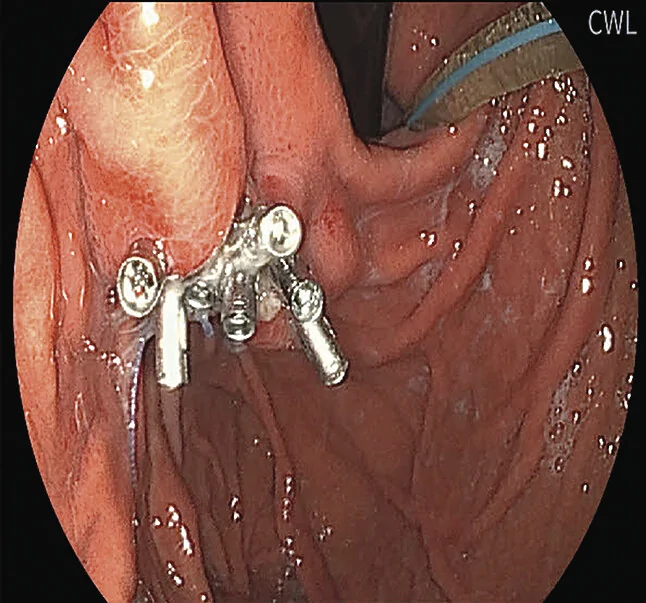
Follow-up endoscopy on postoperative day 3 showing an intact and stable closure with no evidence of slippage.

We report the first application of an absorbable string-loop suture (ALS) for closing a gastric defect following endoscopic full-thickness resection (EFTR). A 56-year-old patient presented with a submucosal tumor on the posterior wall of the gastric fundus ( Fig. 1 ), revealed by endoscopic ultrasound to arise from the muscularis propria with both intraluminal and extraluminal growth. Following detailed clinical consultation, the patient underwent EFTR, exposing typical yellowish retroperitoneal adipose tissue at the defect base ( Fig. 2 ). Video 1 demonstrates the operational process. The basic structure of the ALS was previously described. 1 In this study, the ALS was modified by increasing the diameter and wall thickness of both sheaths, with the inner sheath featuring a tapered distal tip to facilitate knot advancement ( Fig. 3 ). During closure, the loop was clipped to one defect edge while withdrawing the outer sheath. With only the traction thread remaining in the biopsy channel, a second clip was easily passed to fix the loop to the opposite edge. Maintaining traction thread tension while advancing the inner sheath tightened the pre-tied sliding knot, approximating the defect linearly ( Figs. 4 and 5 ). This knot advancement process was smooth and without slippage. Finally, additional clips achieved complete closure. Notably, the traction thread provided external traction to facilitate additional clip placement and was detached directly without scissors. Resection and suturing took 11 and 15 min, respectively. Follow-up endoscopy on postoperative day (POD) 3 confirmed intact closure without slippage ( Fig. 6 ), allowing liquid diet initiation. The patient was discharged on POD 4 without adverse events. Follow-up endoscopy at 3 months confirmed complete mucosal healing. Histology confirmed a completely resected gastrointestinal stromal tumor (representative histopathologic images demonstrated in Video 1 ). Our experience suggests that ALS is a feasible option for effectively closing full-thickness gastric defects; however, further study is warranted.
